# Supplementary material for: Outcome benefits of upfront cytoreductive nephrectomy for patients with metastatic renal cell carcinoma: An analysis of the TriNetX database
Source: PLoS One. 2024 Mar 28;19(3):e0299102. doi: 10.1371/journal.pone.0299102 (PMC10977795; doi:10.1371/journal.pone.0299102)
Supplement: Attachment — Submitted filename: Review Comments_4.pdf [file pone.0299102.s003.pdf]

Review Comments on “Outcome benefits of upfront cytoreductive nephrectomy for patients with metastatic renal cell carcinoma: an analysis of the TrinetX database”

Overall comments:

The authors report potential clinical implications and survival of cytoreductive nephrotomy in patients. The manuscript is a well-written one and describes the limitations of the study too. However, I do have the following suggestions as it would help the reader to understand the findings more conveniently, specifically tables 1 and 2, than how it is now. Because it is difficult now to go through the data and the graphs are crucial to be a part of the scientific data presentation in the manuscript, also required to match the standards of the journal.

1. From Table 1, it is suggested that addition of pie-charts to show the points mentioned in the chart like age, sex, ethnicity, organs, etc. in both TKI and IO, is done. This way it will depict the data in a better presentable manner.
2. From Table 1, similarly a bar graph representation with the statistics included for the BMI and co-morbidities is what is needed.
3. A volcano plot is also preferred to be added to show the distribution of the experimental groups amongst the individuals studied, with and without the treatments.
4. The above-mentioned points need to be addressed for table 2 too.
